# FOXM 1 induces Vasculogenic mimicry in esophageal cancer through β-catenin /Tcf4 signaling

**DOI:** 10.1186/s13000-020-00929-9

**Published:** 2020-02-08

**Authors:** Lili Cheng, Qi Wang, Xiaoying Tao, Yanzi Qin, Qiong Wu, Dafang Zheng, Damin Chai, Yong Zhang, Dongbing Lu, Hongfei Ci, Zhiwei Wang, Jia Ma, Danna Wang, Zenong Cheng, Shiwu Wu, Yisheng Tao

**Affiliations:** 1 Department of Pathology, The First Affiliated Hospital of Bengbu Medical College, Changhuai road 287, Bengbu, Anhui 233000 People’s Republic of China; 2 Department of Pathology, Bengbu Medical College, 2600 Donghai Avenue, Bengbu, Anhui Province China

**Keywords:** Esophageal cancer, FOXM1, β-Catenin, Tcf4, VM, Proliferation, Invasion, Migration

## Abstract

**Objective:**

To investigate the role of FOXM1, β-catenin and TCF4 in esophageal cancer (EC) and their relationship to VM (Vasculogenic Mimicry).

**Methods:**

CCK-8 were performed to examine EC cell proliferation in FOXM1 silenced cells. EC cell migration and invasion were investigated through wound healing and Transwell assays, respectively. The formation of pipe like structures were assessed in 3D cultures. The expression of Foxm1, β-catenin, Tcf4 and E-cadherin were investigated through western blot, RT-qPCR and immunohistochemistry (IHC) staining. The relationship between FOXM1 expression, clinic-pathological features, and overall survival (OS) were further analyzed.

**Results:**

A loss of FOXM1 expression correlated with the OS of ESCC patients. FOXM1 silencing led to a loss of cell growth and suppressed cell migration and invasion in ESCC cells. VM structures were identified in ESCC tissues and human EC cell lines. Mechanistically, FOXM1 was found to promote tumorigenesis through the regulation of β-catenin, Tcf4, and E-cadherin in EC cells, leading to the formation of VM structures.

**Conclusions:**

These findings highlight FoxM1 as a novel therapeutic target in ESCC.

## Background

Esophageal Carcinoma (EC) is an invasive malignancy of the digestive system and the 6th leading cause of tumor-related mortality. EC originates from the mucosa or glands of the esophagus [1, 2]. It is estimated that 17,290 patients in the United States (US) alone were diagnosed with esophageal cancer in 2018, from which 15,850 individuals died [3]. Two major histopathological subtypes of EC exist, including esophageal squamous cell carcinoma (ESCC) and esophageal adenocarcinoma. ESCC is the predominant histological subtype in China, accounting for ~ 90% of cases.

The mechanisms governing EC development remain poorly defined. ESCC is characterized by a high degree of malignancy, metastasis and recurrence, and poor prognosis [4]. Due to the low efficiency of current diagnostics, most ESCC patients present lymph node or distant metastasis at advanced stages. Although the therapeutic effects of surgery combined with radiotherapy and chemotherapy have improved the outcome of ESCC patients, the 5-year OS rates are ≤25% [5, 6]. A further understanding of the molecular mechanisms of ESCC development are required for new diagnostic markers and therapeutic strategies.

FOXM1 is a transcriptional factor in the Forkhead box family, characterized by a conservative DNA-binding domain (winged-helix domain) [7]. FOXM1 transcriptionally regulates the expression of genes that influence cell cycle progression, including p21Cip1, p27Kip1, Cdc25A, and Cdc25B [8, 9]. Accumulating evidence suggests that FOXM1 is overexpressed in various malignancies [10–13]. FOXM1 enhances tumor metastasis in lung carcinoma, breast adenocarcinoma, pancreatic carcinoma, prostate carcinoma, colorectal cancer, ovarian carcinoma, cervical and nasopharyngeal carcinoma, ESCC and glioma [12–20]. Dai and colleagues showed that FOXM1 enhances MMP-2 transcription to promote glioma progression [21]. Li et al. and Zhang et al. also showed that FOXM1 was directly related to the transactivation of vascular endothelial growth factor (VEGF), thereby promoting angiogenesis in gastric tumors and glioma cells [14, 22]. Studies by Yang et al. indicated that the silencing of FOXM1 inhibits epithelial-mesenchymal transformation (EMT) in colon cancer cells [15] and acts as a molecular marker for the prediction of invasion, metastasis and prognosis in colorectal cancer [16].

Wnt signaling is key to cancer development, particularly ESCC. Approximately 90% of ESCC cases show dysregulated Wnt signaling, leading to the nuclear translocation and aggregation of β-catenin through inactivating APC mutations or activating β-catenin mutations, followed by the combination of T-cell factor (TCF) and lymphoid enhancing factor transcription factors (LEF) to activate the transcription of downstream genes, including c-Myc and cyclin D1 [17]. Studies has revealed that FOXM1 regulates is a novel target of Wnt signaling and indispensable for β-catenin/TCF4 transactivation. In glioma, FOXM1 interacts with β-catenin to enhance its nuclear translocation and ability to promote self-renewal and tumorigenesis. In osteosarcoma, FOXM1 and β-catenin directly interact to promote TCF4 binding to the Wnt gene promoter, enhancing Wnt activity [23]. Yoshida et al. found that the deletion of FOXM1 in mice prevented colorectal tumorigenesis through the regulation of β-catenin/TCF4/E-cad signaling and the suppression of EMT [24].

Vasculogenic mimicry (VM) is a novel blood-supply system first proposed in invasive human uveal melanoma and metastatic cutaneous melanoma by Maniotis et al. in 1999. In 2004, Folberg et al. [25] classified VM into two sub-types: tubular and pattern matrix. Tumors modify their blood supply through the ECM to form a vessel like pipeline, representing a tumor microcirculation mode that is independent of vascular endothelial cells [26]. Through VM, tumor tissues can contact host blood vessels for oxygen and nutrition, increasing tumor metastasis and invasion [26–28]. Red blood cells (RBCs) are visible in the VM channel in which no necrotic hemorrhage is evident. Subsequently, fluorescence dyes and activated carbon particles injected into host blood vessels can be detected in the VM channel through laser scanning confocal microscopy [26–28]. Periodic acid-schiff (PAS) positive ECM and tumor cells are arranged in the VM channel with endothelial cells and markers that cannot be detected using light microscopy, transmission electron microscopy or immunohistochemistry [29]. Increasing evidence suggest that cancer stem cells (CSCs) and EMT play a vital role in the tumor vasculature [30–32]. EMT is a temporary and reversible transition from epithelial cells to mesenchymal cells (such as myofibroblasts) that refers to the removal of the differentiated epithelial cells, a loss of polarity, reduced contact with surrounding cells and the extracellular matrix, and the enhanced mobility and migration of epithelial cells. Epithelial marker such as E-cadherin are gradually lost during EMT, whilst mesenchymal phenotypes such as vimentin, N-cadherin, alpha-SMA, Snail and slug increase [33]. In this study, we investigated the roles of FOXM1, β-catenin, TCF4, and E-cadherin in EC to verify the role of FOXM1 in the formation of VM structures. We herein describe a novel anti-angiogenic strategy to target EC tumor vascularization.

## Materials and methods

### Human ESCC samples

From June 2012 to June 2015, 216 cases of paraffin embedded ESCC and normal adjacent esophageal mucosal tissues were collected during surgery in the first affiliated hospital to Bengbu Medical College (Anhui, China). The clinical data of ESCC patients are shown in Table 1. None of the patients accepted radiotherapy or chemotherapy preoperatively. Informed consent was obtained from each patient. The study was approved by the Bengbu Medical College ethics committee.

**Table 1:** Association of VM, FoxM1, β-catenin, TCF4 and E-cad with the clinicopathologic characteristics of ESCC patients (*n* = 216)

variable	VM		X^2^	P	Foxm1		X^2^	P	β-catenin (Nuclear)		X^2^	P	tcf4		X^2^	P	E cad		X^2^	P
	N	P			N	P			N	P			N	P			N	P		
Tissue																				
Con	216	0			183	33			177	39			180	36			38	178		
ESCC	115	101			67	149			73	143			84	132			133	83		
Gender			0.461	0.311			0.413	0.538			0.143	0.426			0.339	0.405			0.326	0.411
Male	67	54			35	86			42	79			43	78			73	48		
Female	48	47			32	63			31	64			41	54			60	35		
Age			1.334	0.166			0.112	0.440			0.204	0.396			0.302	0.365			0.331	0.409
< 60	63	58			37	84			43	78			45	76			71	50		
≥ 60	52	43			30	65			30	65			39	56			62	33		
Anatomical location			0.677	0.42			1.206	0.812			0.404	0.584			0.635	0.702			0.576	0.609
Upper	4	5			3	6			2	7			4	5			5	4		
Middle	70	59			42	87			46	83			49	80			81	48		
Lower	41	37			22	56			25	53			31	47			47	31		
Gross type			0.367	0.596			3.621	0.318			3.345	0.283			3.235	0.303			2.744	0.293
Medullary	37	33			24	46			25	45			28	42			41	29		
Mushroom	52	51			28	75			30	73			32	71			71	32		
Ulcer type	18	15			10	23			14	19			18	15			17	16		
Sclerotic type	8	2			5	5			4	6			6	4			4	6		
Diameter(cm)			0.394	0.572			0.907	0.356			0.556	0.288			0.673	0.535			0.592	0.321
<5	57	51			37	71			41	67			45	63			61	47		
≥ 5	58	50			30	78			32	76			39	69			72	36		
Differentiation			1.714	0.315			1.384	0.301			0.706	0.41			0.811	0.532			0.863	0.501
Well	37	32			17	52			21	48			24	45			43	26		
Mediate	39	32			24	47			24	46			25	46			47	24		
Poor	39	37			26	50			27	49			35	41			43	33		
LNM			76.885	< 0.001			75.328	< 0.001			61.215	< 0.001			65,301	< 0.001			72.325	< 0.001
Yes	14	98			5	107			6	106			5	107			106	6		
No	101	3			62	42			67	37			79	25			27	77		
Serosa infiltration			59.118	< 0.001			62.244	< 0.001			54.511	< 0.001			58,522	< 0.001			59.332	< 0.001
Yes	23	85			11	97			13	95			12	96			97	11		
No	92	16			56	52			60	48			72	36			36	72		
PTNM stage			35.319	< 0.001			46.488	< 0.001			48.027	< 0.001			47,203	< 0.001			47.623	< 0.001
I-II	67	18			63	23			63	22			55	20			21	64		
III-IV	48	83			5	126			10	121			19	112			112	19		

### Reagents

Rabbit monoclonal antibodies against human Foxm1, β-catenin, and Tcf4 were purchased from Abcam, USA. Anti-CD34 antibodies were purchased from Lab Vision Company (USA). Elivison™ plus immunohistochemical assays and diaminobenzedine (DAB) chromogenic reagent kits were purchased from Maixin Biology Company (Fuzhou, China). Periodic Acid Schiff (PAS) reagents were produced in the Department of Clinical Pathology, First Affiliated Hospital of Bengbu Medical College.

### Immunostaining and CD34 + PAS double staining

All paraffin embedded specimens were immobilized in 10% formalin. All sectioned specimens (4 μm thick) were waxed, dehydrated with a gradient alcohol series, and washed in PBS (pH 7.2) for 10 min. Sections were incubated with 3% H_2_O_2_ methanol at room temperature for 10 min to inhibit endogenous peroxidase activity. Samples were boiled in 1.0% citrate (pH 6.0) for 2 min at high pressure for antigen retrieval. Slides were washed in PBS and blocked in goat serum at room temperature for 20 min. Sections were probed with rabbit monoclonal anti-Foxm1(1:250, ab207298, Abcam, USA), anti-β-catenin (1:100, ab32572, Abcam, USA), anti-Tcf4 (1:150, ab217668, Abcam, USA), anti-E -cadherin (1:200, ab1416, Abcam, USA), and anti-CD34 (1:200, ab762, Abcam, USA) primary antibodies overnight at 4 °C, followed by the appropriate secondary antibodies for 30 min. All CD34 labeled specimens were stained with PAS to characterize the glycolic basement membrane of the vascular endothelial cells, in addition to vascular-like (VM) channels [24, 25]. After rinsing with PBS, freshly prepared 3, 3 ‘-diaminobenzidine (DAB) solution was added to the sections for 5 min, and hematoxylin was back-dyed, dehydrated, air-dried and loaded.

### Cell culture

Human Eca109 and Ec9706 cells were obtained from the Chinese Academy of Sciences (Shanghai, China). Cells were cultured in the basic medium supplemented with 10% fetal bovine serum (Gibco; Thermo Fisher Scientific, Inc., Waltham, MA, USA) at 37 °C. Cells were passaged using 0.05% trypsin (Invitrogen, Carlsbad) and seeded into 6- or 96-well plates.

### siRNA transfection

SiRNA targeting FOXM1 (5′-GCUCAUACCUGGUACCUAUTT − 3′ (sense) and 5′-AUAGGUACCAGGUAUGAGCTT- 3′ (antisense), and NC siRNA were purchased from the GenePharma Corporation (Shanghai). EC cells were seeded into 6 well plates and transfected with Lipofectamine 2000 (Invitrogen, Carlsbad, CA, USA) (final siRNA concentration: 50 nM). Cells were divided into three groups: blank (no transfection), NC (cells transfected with NC siRNA fragment), and FOXM1 siRNA groups (cells transfected with FOXM1siRNA fragments) for 24 to 48 h.

### Real-time quantitative PCR

Trizol (Invitrogen; Thermo Fisher Scientific, Inc) was used to isolate total RNA from transfected cells and cDNA was reverse-transcribed using MMLV kits (Promega Corporation). SYBR green Premix Ex Taq II (Qiagen GmbH) and One Step plus RT-PCR systems (Applied Biosystems; Thermo Fisher Scientific, Inc.) were used for qPCR. The ABI Prism 7500 sequence detection system (Applied Biosystems; Thermo Fisher Scientific, Inc.) was used for all PCRs. Reaction conditions were as follows: total volume 25 μl: 10 μl SYBR Premix Ex Taq (2×), 1 μl forward Primer; 1 ul reverse primer; 0.5 μl ROX Reference Dye II (50×)* 3, 2 μl cDNA and 8 μl ddH_2_0. PCR parameters included: initial denaturation at 95 °C for 10 min, followed by 40 cycles of 95 °C for 5 s; 63 °C for 30 s; and 72 °C for 30 s; final extension: 72 °C for 5 min. Relative gene expression was detected using the 2^ΔΔCq^ method (ΔΔCq = ΔCq ESCC ΔCq corresponding normal tissues) [34]. Primers sequences are shown in (Table 2). Gene expression was normalized to GAPDH as an internal standard. All PCRs were performed in triplicate.

**Table 2:** Primer sequences

Genes	Forward primer (5′-3′)	Reverse primer (5′-3′)	Length (bp)
FOXM1	CGTCGGCCACTGATTCTCAAA	GGCAGGGGATCTCTTAGGTTC	96
TCF4	TGCAAAGCCGAATTGAAGATCG	AGAAGGTCCAATGATTCCATGC	131
β-catenin	ATGGACAGTATGCAATGACTCG	TAGCAGACACCATCTGAGGAGA	345
E-cadherin	GAAGTGTCC GAGGACTTTGG	CAGTGTCTCTCCAAATCCGATA	109
GAPDH	CAGCCTCAAGATCATCAGCA	TGTGGTCATGAGTCCTTCCA	106
FOXM1, TCF4, β-catenin, E-cadherin.			

### Western blotting

Cells were lysed in RIPA buffer (Solarbio Technology Co, Ltd., Beijing) and protein concentrations were determined via BCA assay. Proteins (20 μg) were transferred to PVDF membranes (Merck Camille, Darmstadt, Germany) and blocked in 5% skimmed milk in melamine buffer water and Tween 20 at room temperature for 1 h. Membranes were probed with anti-FoxM1 (cat. no: ab207298; 1:1000; Abcam, USA), anti-β-catenin (cat. no: ab32572; 1:5000; Abcam), anti-TCF4 (cat. no: ab217668; 1:10,000; Abcam), anti-E-cadherin (cat. no: ab1416; 1:50; Abcam) and anti-β-Actin (cat. no: ab8226; 1:1000; Abcam) antibodies in 5% skimmed milk overnight at 4 °C. Membranes were labeled with second antibodies (cat. no: ab97080; 1:5000; Abcam) at room temperature for 2 h. Protein bands were visualized using Pierce™ ECL western blotting substrate (Thermo Fisher Scientific, Inc.). GAPDH (cat. no. AC002; 1:1000; ABclonal Biotech Co., Ltd.) was probed as a loading control. Western blot analysis was performed in triplicate. Representative blots are shown.

### Cell viability assays

CCK-8 cell counting kits (Dojindo Molecular Technologies, Inc., Kumamoto, Japan) were used to determine cell viability. Briefly, 1 × 10^4^ EC cells were seeded into 96 well plates and cultured overnight in complete DMEM (Hyclone) at 37 °C. The next day, 10 μl of CCK8 solution was added to each well for 2 h and the absorbance at 450 nm was measured on a micro-plate reader (BioRad Laboratories, Inc., Hercules, CA, USA). In FOXM1 silenced cells, cell numbers were analyzed at 12, 24, 48 and 72 h.

### Wound healing assays

Wound healing assays were used to evaluate the ability of Eca109 cells to migrate. Scratches were introduced into 6-well plates with a 10 μl pipette tip (Eppendorf, Hamburg, Germany) and cells were grown in DMEM plus 1% fetal bovine serum at 5% CO_2_ at 37 °C. Cells in and around the scratch area were imaged at 0 h, 20 h and 30 h. Wound healing was monitored on a light microscope.

### Transwell chamber assays

Transfected EC cells were seeded into Transwell chambers (Costar; Corning Inc., Corning, NY, USA). For invasion assays, chambers were coated with Matrigel (BD, USA) and cells were inoculated in serum free medium (5 × 10^4^ cells). The chamber below included DMEM containing 10% fetal bovine serum. After cell infiltration for 20 h, cells were washed, fixed, and stained with Calcein-AM or crystal violet for 20 min. Invading cells were counted and imaged (Hanrong Company, Shanghai).

### 3D cultures

Preserved matrix glue was dissolved into a liquid state at 4 °C overnight. Micropipette tips and 24-well cell culture plates were precooled on ice, and matrix colloidal solution was added to the cells on ice. Cells were trypsinized into single-cell suspensions at a density of 5 × 10^5^/ml after.

Twelve hour. Tubular structure arrangements and cell integrity then imaged across five visual fields (top, bottom, left, right and center) using an inverted microscope (× 200). The number of tubular structures were counted, and the mean value of each visual field recorded. Experiments were repeated on a minimum of 3 occasions.

### Immunohistochemical staining

IHC was performed by two experienced pathologists using semi-quantitative evaluations. Clinical and follow-up data were assessed through double blind assessments. Ten high-power-fields (HPF) in each ESCC section were used to evaluate intratumoral heterogeneity. Staining was graded according to intensity (0: none; 1: weak; 2: moderate; 3: strong) and extent (1: < 11%; 2: 11–50%; 3: 51–75%; 4: > 75%) and multiplied to derive eventual scores of 0–12. Positive staining was defined as ≥3. For sections positive for Foxm1, β-catenin, Tcf4 and E-cadherin, the final scores of each section were averaged.

### Statistical analysis

All data are expressed as the mean ± stand deviation (SD). A one-way ANOVA followed by Tukey’s post-hoc test was used for inter-group comparisons. A student’s t-test was used to evaluate differences. GraphPad version 5 for Windows (GraphPad Software, USA) was all statistical analysis. Counting data were analyzed using a Chi-square test for group comparisons. Multiple logistic regression analysis was used to determine the factors influencing metastasis. Kaplan-Meier assays with log-rank tests were used for univariate OS analysis. Cox regression models were used for multivariate OS analysis. *P*-values < 0.05 were deemed statistically significant.

## Results

### Association between VM, FOXM1, β-catenin, TCF4 and E-cadherin expression and the clinicopathological features of EC

**Fig. 1:**
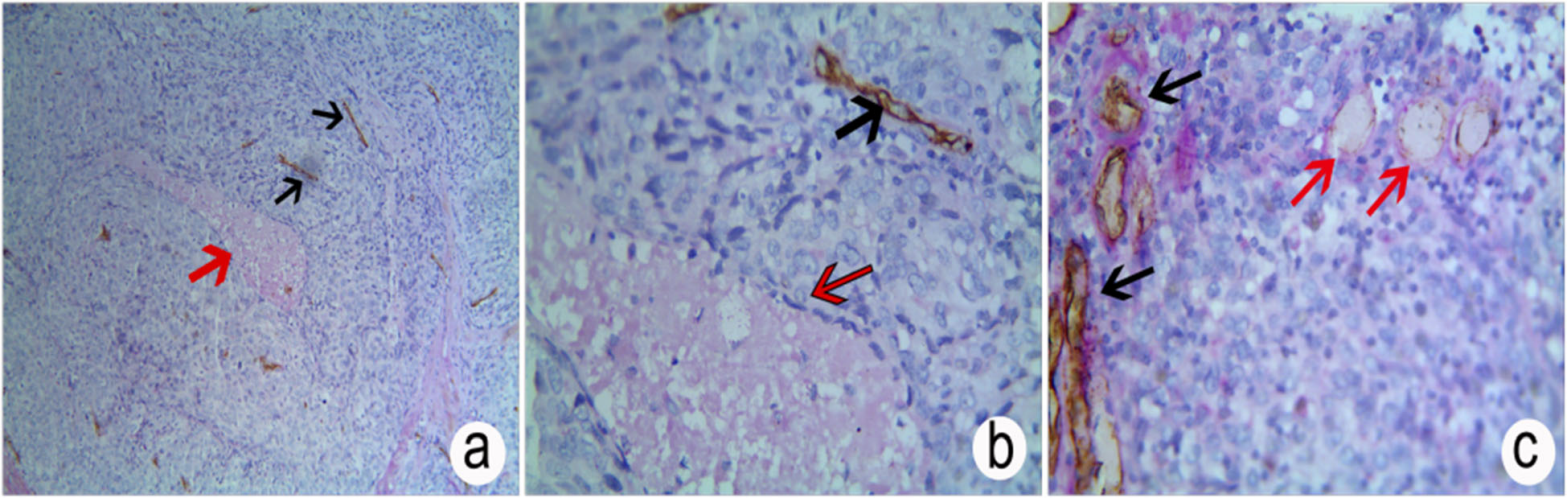
Positive staining of VM and micro-vessels in ESCC through CD34 + PAS double staining. **a** Positive staining of VM and micro-vessels in ESCC (×100); **b** and **c**: Positive staining of VM and micro-vessels in ESCC (×400). Red arrows represent VM; black arrows: micro-vessels

To investigate the role of VM, FOXM1, β-catenin, TCF4 and E-cadherin during ESCC progression, immunohistochemical analysis of both ESCC and corresponding adjacent normal tissues were performed and compared to the clinicopathological features of the patients. VM+ was determined as small vessels with channels in ESCC that were PAS+ but CD34−. The VM structures included circular tubular, straight lines, and networks in the ESCC samples (46.76%; 101/216) that were higher than corresponding adjacent normal tissue (0%; 0/216; *P* < 0.001; Fig. 1a, b, c). The VM in ESCC was related to LNM, serosal invasion, and PTNM stage. No association with patient gender, age, anatomical location, gross morphology, degree of differentiation or tumor diameter were observed (Table 1).

**Fig. 2:**
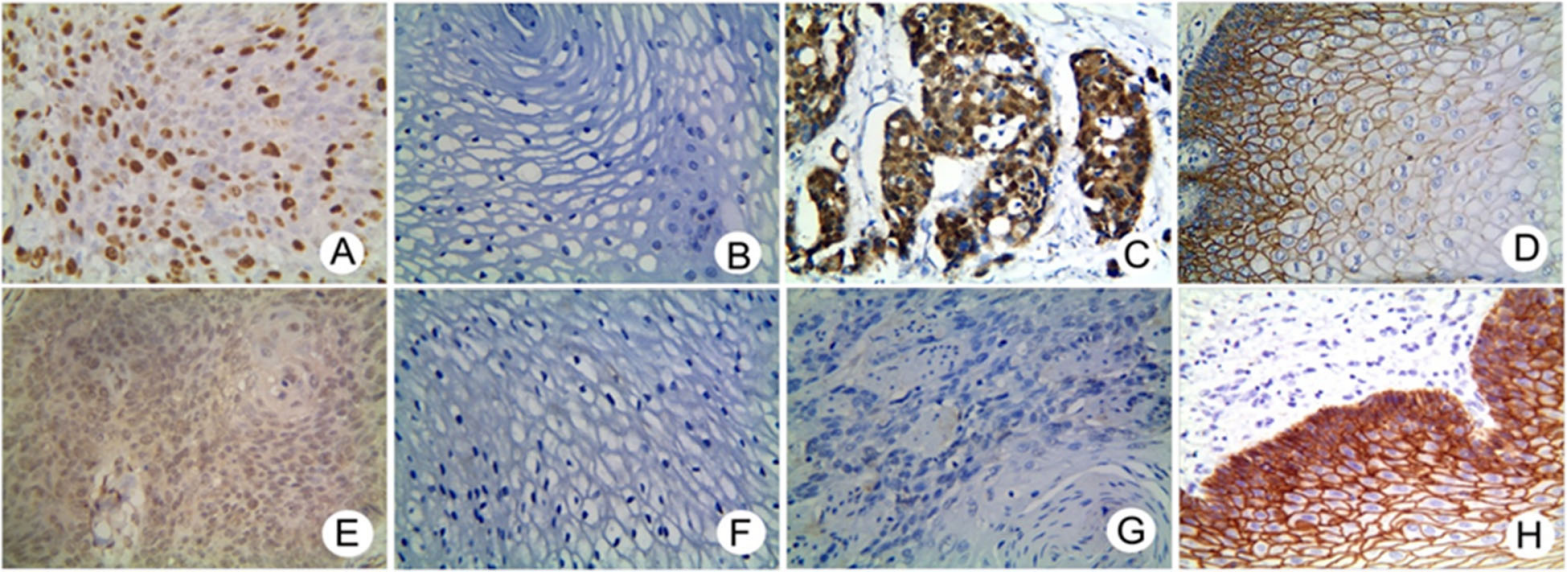
Immunostaining of FoxM1, β-catenin, TCF4 and E-cad in ESCC and control tissues (X 400 magnification). **a** Positive staining of FoxM1 in the nucleus and cytoplasm of tumor cells. **b** Negative staining of FoxM1 in control tissues. **c** Positive staining of β-catenin in the nucleus and cytoplasm of tumor cells. **d** Negative staining of β-catenin in control tissues. **e** Positive staining of TCF4 in the nucleus of tumor cells. **f** Negative staining of TCF4 in control tissues. **g** Negative staining of E-cadherin in tumor cells. **h** Positive staining of E-cadherin in the membrane and cytoplasm of control tissues

As for VM (Fig. 2a), FOXM1+ expression was confined to the nucleus, with higher positivity in ESCC samples (68.98%, 149/216) compared to adjacent normal tissues (15.28%, 33/216; *P* < 0.001; Fig. 2b). The rate of FOXM1+ expression in ESCC was related to LNM, serosal invasion, and PTNM stage, but not with patient gender, age, anatomical location, gross morphology, degree of differentiation, or tumor diameter (Table 3).

**Table 3:** Correlation of VM, FoxM1, β-catenin, TCF4 and E-cad in ESCC (*n* = 216)

Variables	β-catenin (Nuclear)		r	p	TCF4		r	p	E-cad		r	p	VM		r	p
	Negative	Positive			Negative	Positive			Negative	Positive			Negative	Positive		
FoxM1			0.812	*P* < 0.001			0.738	*P* < 0.001			−0.746	*P* < 0.001			0.608	*P* < 0.001
Negative	61	1			62	5			5	21			66	1		
Positive	12	137			22	127			128	21			49	100		
β-catenin							0.896	*P* < 0.001			−0.904	*P* < 0.001			0.670	*P* < 0.001
Negative					73	11			0	73			73	0		
Positive					0	132			133	10			42	101		
TCF4											−0.990	*P* < 0.001			0.729	*P* < 0.001
Negative									1	83			83	1		
Positive									132	0			0	100		
E-cad															−0.740	*P* < 0.001
Negative													32	101		
Positive													83	0		

As is shown in Fig. 2c, β-catenin localized to the nucleus and cytoplasm and its positive expression significantly increased in ESCC tissue (66.20%, 143/216) compared to corresponding adjacent normal tissue (18.06%, 39/ 216; *P* < 0.001; Fig. 2d). The rate of β-catenin + expression was related to lymph node metastasis, serosal infiltration, and PTNM stage. No relationship was observed between β-catenin expression and patient gender, age, anatomical location, gross morphology, degree of differentiation and tumor diameter (Table 1).

As is shown in Fig. 2e, TCF4 + was mainly observed in the nucleus and correlated with the high expression of ESCC tissue (61.11%, 132/216) compared to corresponding adjacent normal tissue (16.67%, 36/ 216; *P* < 0.001; Fig. 2 F). Immunostaining indicated that TCF4 + expression was related to LNM, serosal invasion, and PTNM stage, but not to patient gender, age, anatomical location, gross morphology, the degree of differentiation or tumor diameter (Table 1).

As is shown in Fig. 2g, e-cadherin expression was mainly confined to the membrane and cytoplasm, and positive expression was lower in ESCC tissues (38.43%, 83/ 216) compared to corresponding adjacent normal tissue (82.41%, 178/216; *P* < 0.001; Fig. 2h). Immunostaining demonstrated that E-cad + expression was inversely related to LNM, serosal invasion, and PTNM stage, but not with patient gender, age, anatomical location, gross morphology, the degree of differentiation or tumor diameter (Table 1).

### Association amongst VM and the expression of Foxm1, β-catenin, Tcf4 and E-cad in ESCC

Spearman correlation coefficient analysis showed that the expression of E-cad + negatively correlated with VM (r = − 0.740, *P* < 0.001), Foxm1 (r = − 0.746, *P* = 0.001), β-catenin (r = − 0.904, P < 0.001) and Tcf4 (r = − 0.990, *P* < 0.001). The expression of Foxm1 and VM (r = 0.608, P < 0.001), β-catenin (r = 0.812, P < 0.001) and Tcf4 (r = 0.896, P < 0.001) showed a positive correlation with VM (r = 0.670, *P* < 0.001; Table 3).

#### Survival prognosis

**Fig. 3:**
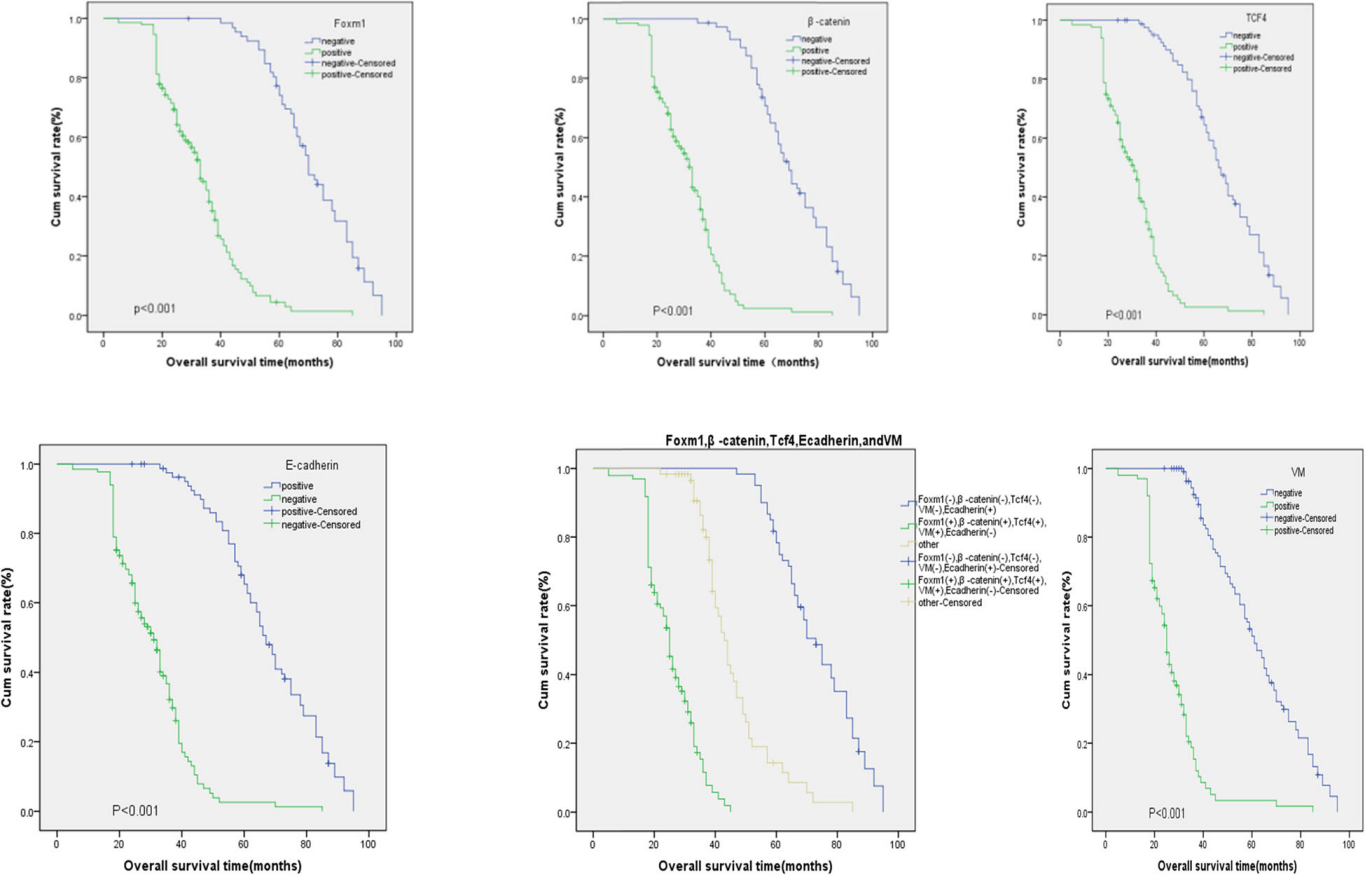
Kaplan-Meier analysis of the survival rates of patients with ESCC. **a** Overall survival of patients in relation to FoxM1 expression (log-rank =121.530, P < 0.001). **b** Overall survival of all patients in relation to β-catenin (log-rank = 132.879, *P* < 0.001). **c** Overall survival of patients in relation to TCF4 (log-rank = 129.901, *P* < 0.001), **d** Overall survival of patients in relation to VM expression (log-rank = 148.841, P < 0.001). **e** Overall survival of patients in relation to E-cadherin (log-rank = 69.208, P < 0.001). In (**a-e**), blue lines represents FoxM1 positive, or β-catenin, TCF4 or VM negative E-cadherin group; green lines: FoxM1 negative, or β-catenin, TCF4 or VM positive E-cadherin group. **f** Overall survival of all patients in relation to FoxM1, β-catenin, TCF4, VM and E-cadherin expression (log-rank = 220.469, *P* < 0.001). Blue lines: patients with negative E-cadherin and positive FoxM1, β-catenin, TCF4, VM expression; green line: patients with positive E-cadherin and negative FoxM1, β-catenin, TCF4, VM; brown line: other positive or negative proteins

The OS was identified as the period from the initial time from surgery to death or at last follow-up. KM survival analysis showed that the median OS (27.526) and OS rates [17.82% (18/101)] in the VM+ group were significantly shorter than the median OS (62.158) and OS rates [72.17% (83/115)] of the VM− group (Log-rank = 148.841, *P* < 0.001). The median OS (33.167) and OS rates [5.37% (8/149)] in the FOXM1+ group were lower than median OS (71.535) and OS rates [49.25% (33/67)] in the FOXM1− group (Log-rank = 121.530, *P* < 0.001). Similarly, the median OS (31.754) and OS rates [14.06% (9/64)] in the β-catenin + group were lower than those of the β-catenin− group (70.317 and 57.34% (81/143), respectively, Log-rank = 132.879, *P* < 0.001). The median OS (31.009) and the OS rates [9.84% (13/132)] of the TCF4 + group were lower than median OS (67.715) and the OS rates [48.81% (41/84)] of the TCF4 − group (Log-rank =129.901, *P* < 0.001). In the E-cad + group, the median OS and the OS rates were 68.091 and 78.95% (105/133), respectively. In the E-cad- group, the OS rates were 31.044 and 15.67% (13/83), respectively (Log-rank = 131.574, *P* < 0.001) (Fig. 3).

Multivariate analysis indicated that VM, Foxm1, β-catenin, Tcf4, E-cadherin, LNM, serosal infiltration, and PTNM stage were independent prognostic factors for ESCC (Table 4).

**Table 4:** Multivariate survival analysis of patients with ESCC (*n* = 216)

Variables	B	SE	Wald	df	Sig.	Exp(B)	95.0%CI	
							Down	Up
Gender	0.035	0.213	0.031	1	0.932	1.103	0.681	1.582
Age	0.042	0.228	0.033	1	0.855	1.042	0.667	1.629
Site	0.207	0.187	1.225	1	0.268	1.230	0.852	1.776
Gross morphology	−0.600	0.134	0.201	1	0.654	0.942	0.725	1.224
Size	0.143	0.211	0.464	1	0.496	1.154	0.764	1.744
Differentiation	0.016	0.142	0.013	1	0.909	1.016	0.769	1.343
LNM	0.299	0.547	1.004	1	0.043	1.136	0.332	1.481
Serosa invasion	−0.475	0.631	0.134	1	0.037	1.326	0.237	1.664
PTNM	−0.032	0.32	0.010	1	0.020	0.968	0.157	1.813
FoxM1	2.260	0.539	17.595	1	0.001	9.582	3.333	27.546
β-catenin	2.373	0.825	8.271	1	0.004	10.732	2.129	54.085
TCF4	2.221	0.492	21.485	1	0.003	10.133	3.022	43.132
E-cad	−1.703	0.381	19.811	1	0.022	0.465	0.154	1.932
VM	2.097	0.42	24.943	1	0.001	8.14	3.575	18.535

### FoxM1 analysis

**Fig. 4:**
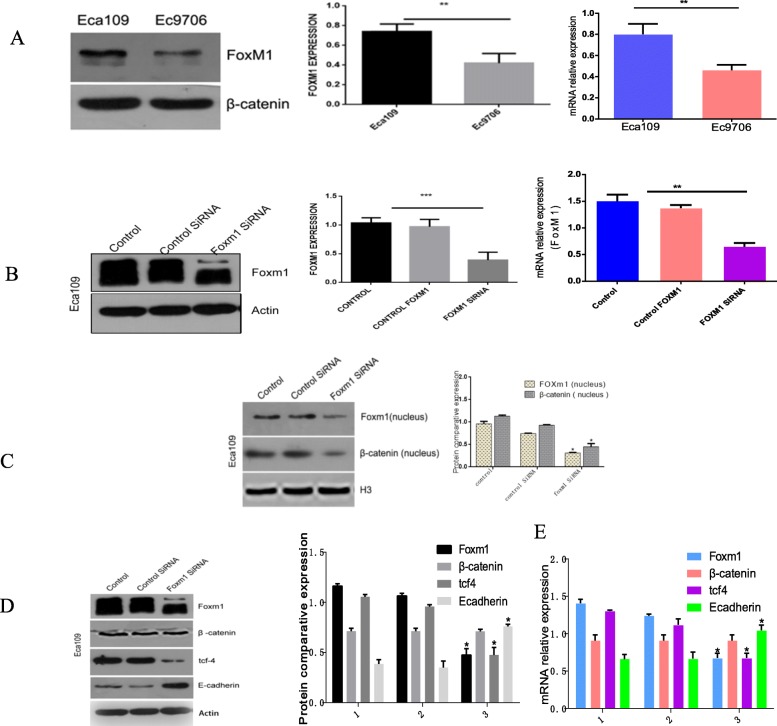
**a** Top panel: Western blotting and RTq-PCR analysis of Foxm1 expression in Eca109 and Ec9706 cells, respectively. Bottom panel: Quantitative analysis *P<0.05; **b** Top panel: Foxm1 expression of three groups of EC cells following FoxM1 silencing. Bottom panel: Quantitative data for the top panel. *P<0.05; **c** Top panel: Western blotting to detect Foxm1 and β-catenin expression in the nuclei of the three groups EC cells after FoxM1 silencing. Bottom panel: Quantitative data for the top panel. *P<0.05; **d** Top panel: Western blotting to detect the expression of Foxm1, β-catenin, tcf4, and E-cad in the three groups of EC cells after FoxM1 silencing. Bottom panel: Quantitative data of the top panel. *P<0.05; **e** Top panel: RT-PCR to measure the mRNA levels of Foxm1, β-catenin, tcf4 and E-cad in the three groups of EC cells after FoxM1 silencing. Bottom panel: quantitative data for the top panel. *P<0.05

The expression of FoxM1 in Ec9706 cells was ~ 46% lower than that of Eca109 cells (*P* < 0.05, Fig. 4a). Eca109 cells were therefore selected for subsequent analysis. Cells were transfected with FoxM1-siRNA and silencing was confirmed at both the mRNA and protein levels (*P* < 0.05, Fig. 4b).

### FoxM1 silencing inhibits the proliferation of EC cells

**Fig. 5:**
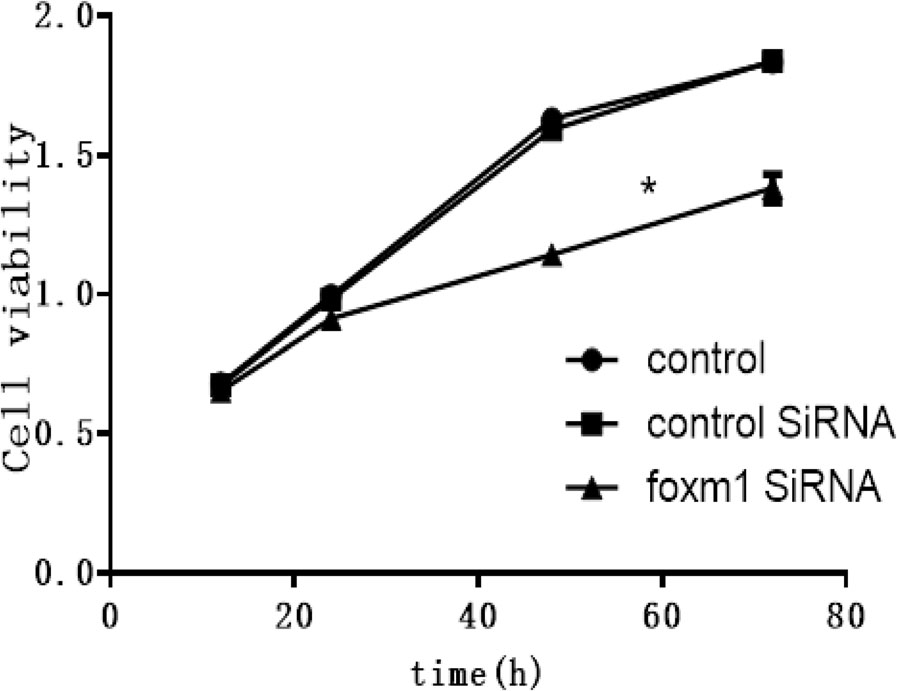
CCK-8 assays across the three groups at 12 h, 24 h, 48 h and 72 h after FoxM1 silencing. *P<0.05 vs control

CCK-8 assays were used to investigate the effects of FoxM1 silencing in Eca109 cells. The results showed that the OD absorbance values in the FoxM1-siRNA group increased compared to NC groups (*P* < 0.05, Fig. 5) suggesting that FoxM1 silencing inhibited cell proliferation. No differences were observed between NC and blank control groups (*p* > 0.05).

### FoxM1 silencing inhibits the invasion and migration of EC cells

**Fig. 6:**
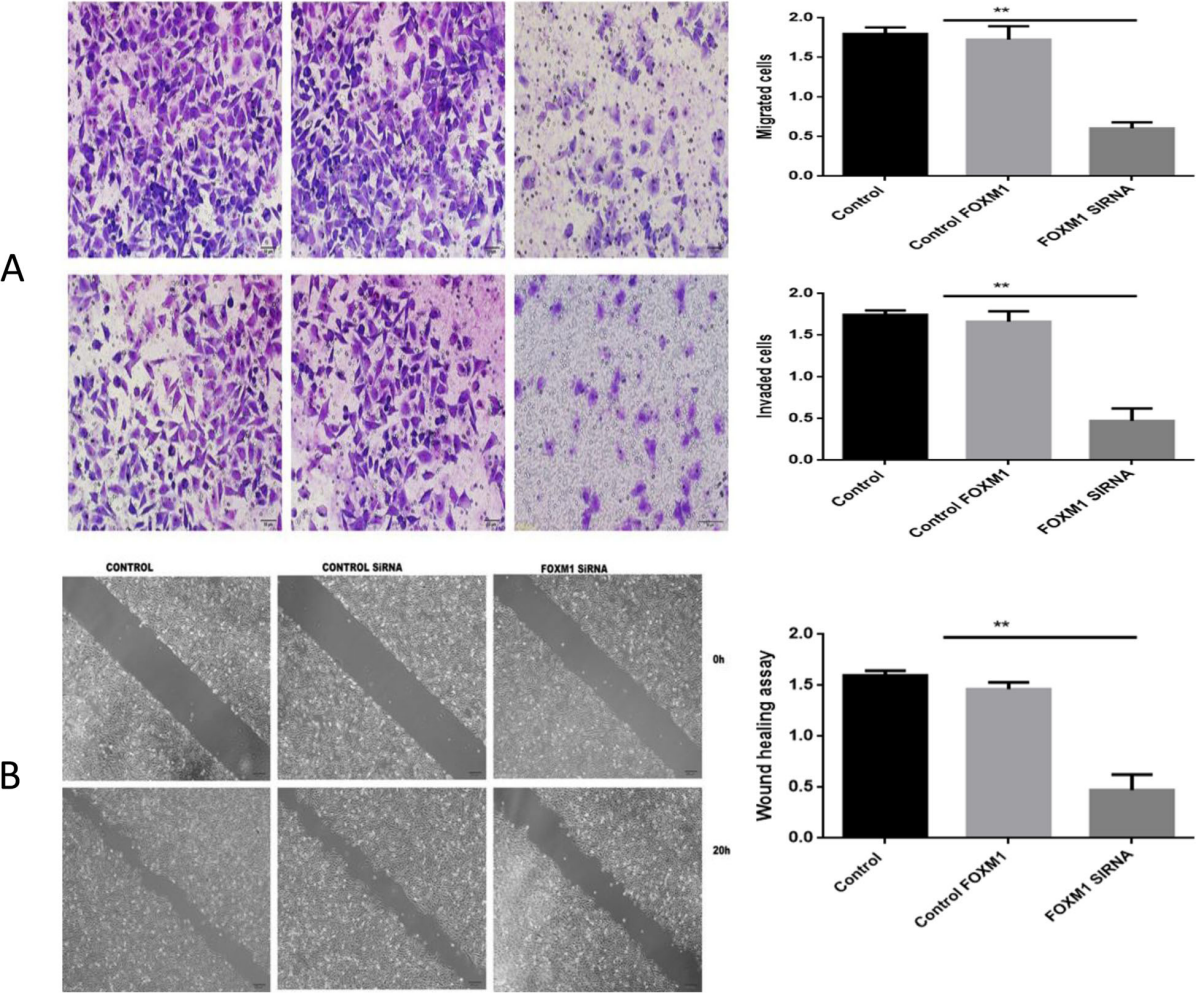
**a** Top panel: Migration and invasion assays following FoxM1 silencing. Bottom panel: Quantitative results are shown for panel. * *P<0.01 vs control (× 200); **b** Top panel: Wound healing assays to measure the migratory capacity in three groups of EC cells after FoxM1 silencing. Bottom panel: quantitative analysis. **P<0.01 vs control

Transwell migration assays showed that the number of cells penetrating the cell membrane (98.00 ± 10.97) in the FOXM1-silenced groups were lower than those of the NC (296.00 ± 6.66) or blank control groups (283.70 ± 12.41). Cell migration also significantly declined (*p* < 0.05, Fig. 6). No significant differences were observed between NC and blank control groups (*P* > 0.01). The levels of cell membrane penetration (76.33 ± 2.40) were lower in the FoxM1-silenced group compared to NC (270.67 ± 1.45) and blank control groups (260.33 ± 3.18). Cell invasion ability also significantly declined (*P* < 0.05, Fig. 6). No significant differences were observed between NC and blank groups (*P* > 0.01). Wound healing assays showed that FoxM1 silencing led to slower wound closure rates compared to the control group (*P* < 0.05, Fig. 6).

### Effects of Foxm1 silencing on β-catenin, Tcf4, and E-cadherin expression

The expression of Wnt related proteins in each group were investigated by western blot and qRT-PCR. The results showed that the expression FoxM1 and Tcf4 in FoxM1 silenced groups were significantly lower than blank- and negative control groups (*P* < 0.05, Fig. 4d-e). The expression of E-cadherin in the FoxM1-siRNA group was higher than that of blank- or negative control groups (*P* < 0.05). No significant differences in total β-catenin expression were observed (*P* > 0.05). The nuclear localization of β-catenin decreased in the FoxM1-siRNA group (*P* < 0.01, Fig. 4c). These findings confirmed that FoxM1 silencing prevent the EMT of tumor cells.

### Assessment of 3D tubular structures

**Fig. 7:**
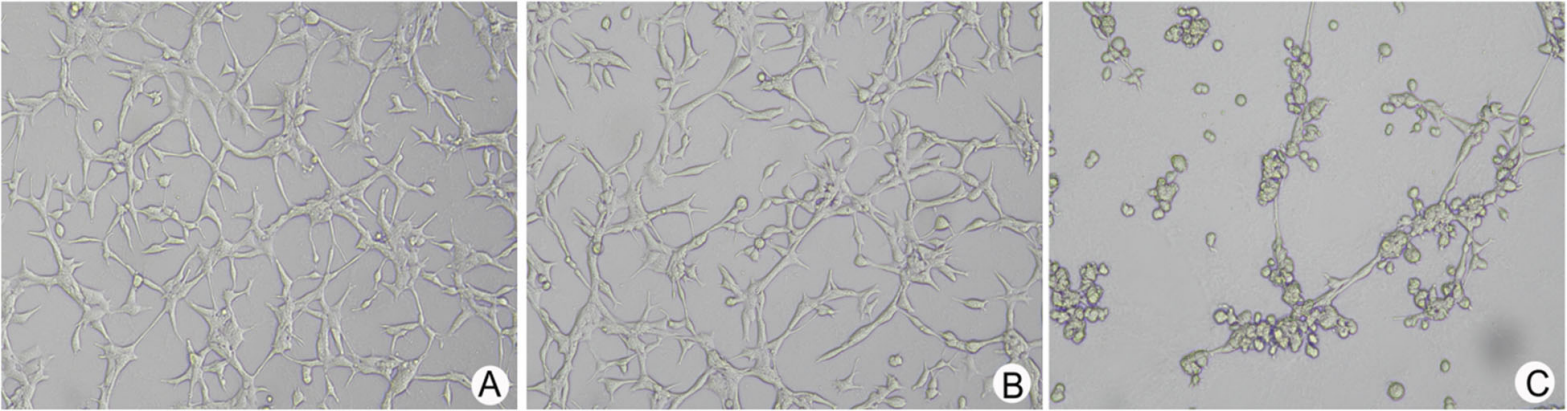
Top panel: Three-dimensional cultures to quantify pipeline formation. Bottom panel: Quantitative data. (**a**: control group; **b**: control SiRNA group; **c**: FoxM1 siRNA group) **P<0.01 vs control

We assessed 3D tubular structures to investigate cell pipeline formation. Blank- and NC groups could simulate the characteristics of vascular endothelial cells and could connect on the matrix gel. After 12 h, a typical vascular network structure was formed, with single multi-ring connections. However, in the FoxM1-siRNA group, tube was significantly inhibited, and annular structures were broken, most of which were incompletely closed linear structures. The quantity of tubular structures in the FOXM1-SiRNA group were also significantly lower than the control group (14.0 ± 1.2 vs. 30.0 ± 1.2, 27.7 ± 1.5, *P* < 0.05, Fig. 7).

## Discussion

Esophageal cancer (EC) is a prevailing tumor of the digestive system and a primary cause of cancer-related mortality. The high heterogeneity of EC has led to a paucity of available biomarkers. To develop novel approaches to diagnosis and treatment, an improved understanding of the mechanism(s) of ESCC are required. Accumulating evidence demonstrates that FOXM1 is involved in the occurrence and development of the VM structures observed in EC, but the molecular mechanisms underlying these effects have not been elucidated. In this study, the outcome of candidate biomarkers were comprehensively evaluated to ensure their effectiveness.

Highly invasive tumors have pipe-like structures, which are independent of vascular endotheliocytes and are directly formed by tumor cells, permitting contact with host blood vessels through their own deformation and ECM reconstruction to gain a blood source [26, 35]. In this study, VM’ structures were observed in 101 of 216 patients with ESCC, but not in corresponding adjoining normal tissue, confirming the presence of VM in ESCC. Upon PAS and CD34 double staining, VM channels form in straight lines, curves or branches, and only one layer of PAS-positive substances can be observed between tumor cells and the blood flow, with no surrounding endotheliocytes. This leads to tumors more prone to recurrence and migration causing poor outcomes and high mortality rates [36–39]. This also demonstrates that VM is closely related to LNM, serosal invasion and the PTNM stage of ESCC. Survival analysis further indicated that the negative expression of VM in ESCC patients correlates with longer survival times. We conclude that tumors with VM’ structures show poor differentiation, low clinical staging, and are more prone to lymph node metastasis [40–43]. FOXM1+ expression was higher in ESCC tissues than control tissues. The rates of FOXM1+ expression in ESCC were related to LNM, serosal invasion, and PTNM stage. Survival analysis further demonstrated that FOXM1 negative groups of ESCC patients had longer survival times than positive expression groups. Β-catenin + expression was also significantly higher in ESCC tissues than in control tissue. The rates of β-catenin + expression positively associated with LNM, serosal invasion, and PTNM stage. Survival analysis also indicated that ESCC patients in the β-catenin− group had longer survival times than the positive expression group. No relationship between β-catenin + expression and patient gender, age, anatomical location, gross morphology, the degree of differentiation and tumor diameter were observed. TCF4 + expression was higher in ESCC vs. control specimens. TCF4 + expression was also related to LNM, serosal invasion, and PTNM stage. Survival analysis indicated that the TCF4 negative expression group of ESCC patients had longer survival times than the positive expression group. E-cadherin + expression was significantly lower in ESCC tissue compared to control tissue and inversely related to LNM, PTNM stage and serosal infiltration. Survival analysis also showed that ESCC patients in the E-cadherin + group had longer survival times than the negative expression group. Multivariate analysis identified that LNM, serosa invasion, PTNM stages, VM, FoxM1, β-catenin, Tcf4 and E-cadherin were all independent predictors of ESCC. In clinical practice, LNM, PTNM stages and serosal invasion are considered to predict patient outcomes. In the same way, we identified a positive correlation between VM, FoxM1, β-catenin and Tcf4 and a negative relation with E-cadherin. These results indicate that the upregulation of VM, FoxM1, β-catenin, Tcf4 and the inhibition of E-cadherin plays a key role in the malignant transformation of EC cells in the esophagus, and in the progression, metastasis and invasion of ESCC. The results were in agreement with previous studies [44–46].

Increasing evidence has demonstrated that FoxM1 accelerates tumor invasion and migration by regulating eukaryotic extension factor 2 kinase eEF2 in triple negative breast cancer [47]. MicroRNA 630 (mi-RNA-630) inhibits the invasion and migration of gastric cancer cells by targeting FoxM1 [48]. FoxM1 increases the invasion and migration of colorectal cancer by binding to the HSPA5 promoter. Here, we found that the FoxM1 silencing inhibited cell growth and invasion, indicating that FoxM1 functions as a tumor promoter in ESCC. In addition, the network tube structure of FoxM1-siRNA groups was significantly lower than negative control or blank control groups. This suggests a positive correlation between FoxM1 and VM in EC.

The question remains as to how FOXM1 regulates tumor cell growth, invasion and the formation of VM structures in ESCC? The dysregulation of Wnt signaling is closely related to the biological behavior of various tumors. Aberrant activation of Wnt inhibits FoxM1 phosphorylation through the GSK3-Axin complex and interacts with the deubiquitinating enzyme USP5, leading to the deubiquitination of FoxM1 and the maintenance of its stability. FoxM1 accumulates in the nucleus and promotes the recruitment of β-catenin to target gene promoters of the Wnt pathway, leaving the β-catenin/TCF4 complex non-competitively inhibited by ICAT, thereby activating Wnt signaling [49]. Studies have shown that in osteosarcomas, FoxM1 and β-catenin directly bind to the cytoplasm and nucleus, enhancing the nuclear transcription of β-catenin, promoting its binding with TCF4 on the Wnt target gene promoter, enhancing the activity of the Wnt pathway [23]. Jiang et al. [50] also reported that the down-regulation of β-catenin inhibits the proliferation of human nasopharyngeal carcinoma cells and reduces their invasion ability. Blocking the translocation of β-catenin into the nucleus using CGP57380 to inhibit the MNK-EIF4e axis inhibits the proliferation and progression of nasopharyngeal carcinoma cells, and reduces their ability to migrate, invade and metastasize [51]. We further showed that when FoxM1 was silenced, the expression of Tcf4 decreased, whilst the expression of E-cad increased. Despite the comparable levels of β-catenin, its nuclear translocation markedly decreased in the FoxM1-siRNA group. This further demonstrated that FoxM1 activates β-catenin by activating Wnt signaling, enhancing the activation of the TCF/LEF promoter, leading to a loss of E-cadherin expression, intercellular adhesion ability, and the formation of VM structures.

## Conclusions

This study highlights the influence of FOXM1 on the occurrence of ESCC that results in the formation of VM’ structures through the β-catenin/TCF4 axis in ESCC.

## Data Availability

All data are available from the corresponding author upon reasonable request.

## Abbreviations

EMT: Epithelial-mesenchymal transition
ESCC: Esophageal squamous cell carcinoma
FOXM1: Forkhead box protein M1
LEF: lymphoid enhancing factor transcription factors
TCF-4: T-cell factor-4 isoform
VM: Vasculogenic Mimicry

## References

[1] Bray F, Ferlay J, Soerjomataram I, Siegel RL, Torre LA, Jemal A (2018). Global cancer statistics 2018: GLOBOCAN estimates of incidence and mortality worldwide for 36 cancers in 185 countries. CA Cancer J Clin.

[2] Siegel RL, Miller KD, Jemal A (2018). Cancer statistics, 2018. CA Cancer J Clin.

[3] Chen W, Zheng R, Baade PD, Zhang S, Zeng H, Bray F (2016). Cancer statistics in China, 2015. CA Cancer J Clin.

[4] Pennathur A, Gibson MK, Jobe BA, Luketich JD (2013). Oesophageal carcinoma. Lancet.

[5] Zeng H, Zheng R, Zhang S, Zuo T, Xia C, Zou X (2015). Esophageal cancer statistics in China, 2011: estimates based on 177 cancer registries. Thoracic Cancer.

[6] Kumagai K, Rouvelas I, Tsai JA, Mariosa D, Lind PA, Lindblad M (2015). Survival benefit and additional value of preoperative chemoradiotherapy in resectable gastric and gastro-oesophageal junction cancer: A direct and adjusted indirect comparison meta-analysis. Eur J Surg Oncol.

[7] Gartel AL (2010). A new target for proteasome inhibitors: FoxM1. Expert Opin Investig Drugs.

[8] Kalin TV, Ustiyan V, Kalinichenko VV (2011). Multiple faces of FoxM1 transcription factor. Cell Cycle.

[9] Teh M-T (2012). FOXM1 coming of age: time for translation into clinical benefits?. Front Oncol.

[10] Ahmad A, Wang Z, Kong D, Ali S, Li Y, Banerjee S (2009). FoxM1 down-regulation leads to inhibition of proliferation, migration and invasion of breast cancer cells through the modulation of extra-cellular matrix degrading factors. Breast Cancer Res Treat.

[11] Kim I-M, Ackerson T, Ramakrishna S, Tretiakova M, Wang IC, Kalin TV (2006). The Forkhead box m1 transcription factor stimulates the proliferation of tumor cells during development of lung Cancer. Cancer Res.

[12] Kong X, Li L, Li Z, Le X, Huang C, Jia Z (2013). Dysregulated expression of FOXM1 isoforms drives progression of pancreatic Cancer. Cancer Res.

[13] Zhang HG, Xu XW, Shi XP, Han BW, Li ZH, Ren WH (2015). Overexpression of forkhead box protein M1 (FOXM1) plays a critical role in colorectal cancer. Clin Transl Oncol.

[14] Zhang Y, Zhang N, Dai B, Liu M, Sawaya R, Xie K (2008). FoxM1B transcriptionally regulates vascular endothelial growth factor expression and promotes the angiogenesis and growth of Glioma cells. Cancer Res.

[15] Yang K, Jiang L, Hu Y, Yu J, Chen H, Yao Y (2015). Short hairpin RNA- mediated gene knockdown of FOXM1 inhibits the proliferation and metastasis of human colon cancer cells through reversal of epithelial-to-mesenchymal transformation. J Exp Clin Cancer Res.

[16] Chu X-Y, Zhu Z-M, Chen L-B, Wang J-H, Su Q-S, Yang J-R (2012). FOXM1 expression correlates with tumor invasion and a poor prognosis of colorectal cancer. Acta Histochem.

[17] Behrens J, Lustig B (2004). The Wnt connection to tumorigenesis. Int J Dev Biol.

[18] Xu N, Jia D, Chen W, Wang H, Liu F, Ge H (2013). FoxM1 is associated with poor prognosis of non-small cell lung Cancer patients through promoting tumor metastasis. PLoS One.

[19] Wen N, Wang Y, Wen L, Zhao S-H, Ai Z-H, Wang Y (2014). Overexpression of FOXM1 predicts poor prognosis and promotes cancer cell proliferation, migration and invasion in epithelial ovarian cancer. J Transl Med.

[20] Liu M, Dai B, Kang S-H, Ban K, Huang F-J, Lang FF (2006). FoxM1B is overexpressed in human Glioblastomas and critically regulates the Tumorigenicity of Glioma cells. Cancer Res.

[21] Dai B, Kang SH, Gong W, Liu M, Aldape KD, Sawaya R (2007). Aberrant FoxM1B expression increases matrix metalloproteinase-2 transcription and enhances the invasion of glioma cells. Oncogene..

[22] Li Q, Zhang N, Jia Z, Le X, Dai B, Wei D (2009). Critical role and regulation of transcription factor FoxM1 in human gastric Cancer angiogenesis and progression. Cancer Res.

[23] Zhang W, Duan N, Zhang Q, Song T, Li Z, Zhang C (2017). DNA methylation mediated Down-regulation of miR-370 regulates cell growth through activation of the Wnt/β-catenin signaling pathway in human osteosarcoma cells. Int J Biol Sci.

[24] Yoshida Y, Wang IC, Yoder HM, Davidson NO, Costa RH (2007). The Forkhead box M1 transcription factor contributes to the development and growth of mouse colorectal Cancer. Gastroenterology..

[25] Folberg R, Maniotis AJ (2004). Vasculogenic mimicry. APMIS..

[26] Maniotis AJ, Folberg R, Hess A, Seftor EA, Gardner LMG, Pe'er J (1999). Vascular Channel formation by human melanoma cells in vivo and in vitro: Vasculogenic mimicry. Am J Pathol.

[27] Zhang S, Zhang D, Sun B (2007). Vasculogenic mimicry: current status and future prospects. Cancer Lett.

[28] Zhang S, Zhang D, Wang Y, Zhao W, Guo H, Zhao X (2006). Morphologic research of microcirculation patterns in human and animal melanoma. Med Oncol.

[29] Sun B, Zhang S, Zhao X, Zhang W, Hao X (2004). Vasculogenic mimicry is associated with poor survival in patients with mesothelial sarcomas and alveolar rhabdomyosarcomas. Int J Oncol.

[30] Liu TJ, Sun BC, Zhao XL, Zhao XM, Sun T, Gu Q (2012). CD133+ cells with cancer stem cell characteristics associates with vasculogenic mimicry in triple-negative breast cancer. Oncogene..

[31] Sun T, Zhao N (2009). Zhao X-l, Gu Q, Zhang S-w, Che N, et al. expression and functional significance of Twist1 in hepatocellular carcinoma: its role in vasculogenic mimicry. Hepatology..

[32] Zhang Y, Sun B, Zhao X, Liu Z, Wang X, Yao X (2013). Clinical significances and prognostic value of cancer stem-like cells markers and vasculogenic mimicry in renal cell carcinoma. J Surg Oncol.

[33] Zhao D, Besser AH, Wander SA, Sun J, Zhou W, Wang B (2015). Cytoplasmic p27 promotes epithelial–mesenchymal transition and tumor metastasis via STAT3-mediated Twist1 upregulation. Oncogene..

[34] Livak KJ, Schmittgen TD (2001). Analysis of relative gene expression data using real-time quantitative PCR and the 2−ΔΔCT method. Methods..

[35] Barinaga M (1999). New type of blood vessel found in tumors. Science..

[36] Yue W-Y, Chen Z-P (2005). Does Vasculogenic mimicry exist in astrocytoma?. J Histochem Cytochem.

[37] Shen Y, Quan J, Wang M, Li S, Yang J, Lv M (2017). Tumor vasculogenic mimicry formation as an unfavorable prognostic indicator in patients with breast cancer. Oncotarget..

[38] Guo Q, Yuan Y, Jin Z, Xu T, Gao Y, Wei H (2016). Association between tumor Vasculogenic mimicry and the poor prognosis of gastric Cancer in China: an updated systematic review and meta-analysis. Biomed Res Int.

[39] Pulford E, Hocking A, Griggs K, McEvoy J, Bonder C, Henderson DW (2016). Vasculogenic mimicry in malignant mesothelioma: an experimental and immunohistochemical analysis. Pathology..

[40] Wu S, Yu L, Wang D, Zhou L, Cheng Z, Chai D (2012). Aberrant expression of CD133 in non-small cell lung cancer and its relationship to vasculogenic mimicry. BMC Cancer.

[41] Zhu B, Zhou L, Yu L, Wu S, Song W, Gong X (2017). Evaluation of the correlation of vasculogenic mimicry, ALDH1, KAI1 and microvessel density in the prediction of metastasis and prognosis in colorectal carcinoma. BMC Surg.

[42] Yu L, Zhu B, Wu S, Zhou L, Song W, Gong X (2017). Evaluation of the correlation of vasculogenic mimicry, ALDH1, KiSS-1, and MACC1 in the prediction of metastasis and prognosis in ovarian carcinoma. Diagn Pathol.

[43] Yang Y-G, Sari IN, Zia MF, Lee SR, Song SJ, Kwon HY (2016). Tetraspanins: spanning from solid tumors to hematologic malignancies. Exp Hematol.

[44] Chung Y, Law S, Kwong DLW, Luk JM (2010). Serum soluble E-cadherin is a potential prognostic marker in esophageal squamous cell carcinoma. Dis Esophagus.

[45] Research Committee on Malignancy of Esophageal Cancer JSfED (2001). Prognostic significance of CyclinD1 and E-Cadherin in patients with esophageal squamous cell carcinoma: multiinstitutional retrospective analysis1 1No competing interests declared. J Am College of Surgeons.

[46] Zhao X-J (2003). Expression of e-cadherin and β-catenin in human esophageal squamous cell carcinoma: relationships with prognosis. World J Gastroenterol.

[47] Hamurcu Z, Ashour A, Kahraman N, Ozpolat B (2016). FOXM1 regulates expression of eukaryotic elongation factor 2 kinase and promotes proliferation, invasion and tumorgenesis of human triple negative breast cancer cells. Oncotarget..

[48] Feng J, Wang X, Zhu W, Chen S, Feng C (2017). MicroRNA-630 suppresses epithelial-to-mesenchymal transition by regulating FoxM1 in gastric cancer cells. Biochem Mosc.

[49] Chen Y, Li Y, Xue J, Gong A, Yu G, Zhou A (2016). Wnt-induced deubiquitination FoxM1 ensures nucleus β-catenin transactivation. EMBO J.

[50] Jiang R, Niu X, Huang Y, Wang X (2016). β-Catenin is important for cancer stem cell generation and tumorigenic activity in nasopharyngeal carcinoma. Acta Biochim Biophys Sin.

[51] Wang W, Wen Q, Luo J, Chu S, Chen L, Xu L (2017). Suppression of β-catenin nuclear translocation by CGP57380 decelerates poor progression and potentiates radiation-induced apoptosis in nasopharyngeal carcinoma. Theranostics..

